# Incidence and predictors of loss to follow-up among HIV-positive adults in northwest Ethiopia: a retrospective cohort study

**DOI:** 10.1186/s41182-020-00266-z

**Published:** 2020-09-14

**Authors:** Molla Yigzaw Birhanu, Cheru Tesema Leshargie, Animut Alebel, Fasil Wagnew, Melkamu Siferih, Tsige Gebre, Getiye Dejenu Kibret

**Affiliations:** 1 Department of Public Health, College of Health Sciences, Debre Markos University, P.O. Box 269, Debre Markos, Ethiopia; 2 Department of Gynecology and Obstetrics, School of Medicine, Debre Markos University, Debre Markos, Ethiopia

**Keywords:** ART, Debre Markos, HIV/AIDS, Loss to follow-up, Predictors

## Abstract

**Background:**

Despite the rapid expansion of antiretroviral therapy services, ‘loss to follow-up’ is a significant public health concern globally. Loss to follow-up of individuals from ART has a countless negative impact on the treatment outcomes. There is, however, limited information about the incidence and predictors of loss to follow-up in our study area. Thus, this study aimed to determine the incidence rate and predictors of loss to follow-up among adult HIV patients on ART.

**Methods:**

A retrospective cohort study was undertaken using 484 HIV patients between January 30, 2008, and January 26, 2018, at Debre Markos Referral Hospital. All eligible HIV patients who fulfilled the inclusion criteria were included in this study. Data were entered into Epi-data Version 4.2 and analyzed using STATA^TM^ Version 14.0 software. The Nelson-Aalen cumulative hazard estimator was used to estimate the hazard rate of loss to follow-up, and the log-rank test was used to compare the survival curve between different categorical variables. Both bivariable and multivariable Cox-proportional hazard regression models were fitted to identify predictors of LTFU.

**Results:**

Among a cohort of 484 HIV patients at Debre Markos Referral Hospital, 84 (17.36%) were loss their ART follow-up. The overall incidence rate of loss to follow-up was 3.7 (95% CI 3.0, 5.0) per 100 adult-years. The total LTFU free time of the participants was 2294.8 person-years. In multivariable Cox-regression analysis, WHO stage IV (AHR 2.8; 95% CI 1.2, 6.2), having no cell phone (AHR 1.9; 95% CI 1.1, 3.4), and rural residence (AHR 0.6; 95% CI 0.37, 0.99) were significant predictors of loss to follow-up.

**Conclusion:**

The incidence of loss to ART follow-up in this study was low. Having no cell phone and WHO clinical stage IV were causative predictors, and rural residence was the only protective factor of loss to follow-up. Therefore, available intervention modalities should be strengthened to mitigate loss to follow-up by addressing the identified risk factors.

## Plain English summary

Loss to follow-up is a serious public health concern throughout the world. In Ethiopia, there is limited information regarding incidence and predictors of loss to follow-up amongst adult HIV patients on ART. Therefore, this retrospective cohort study estimates the incidence and predictors of loss to follow-up among adult HIV patients on ART at Debre Markos referral hospital. About 484 samples were included in the study. Data analysis was carried out using STATA Version 14.0 statistical software. The cox-proportional hazard regression model was used to identify the predictors of loss to follow-up. In this study, the incidence of loss to follow-up in this study was high. Having no cell phone, rural residence, and WHO clinical stage IV were the predictors of loss to follow-up

## Background

*The* acquired immunodeficiency syndrome (AIDS) pandemic remains the most serious of infectious *disease obstacles* to *public* health [1]. On the other hand, ART suppress the viral multiplication, and enhance the patient’s quality of life [2–4]. Since according to the World Health Organization (WHO) 2018 and UNAIDS 2019 global HIV/AIDS epidemic report, ART had a significant contribution to new HIV infection fell by 37% and HIV/AIDS-related death by 45% correspondingly [5, 6]. In addition, the risk of HIV transmission from HIV patients who had good ART adherence with regular follow-up to their uninfected sexual partners was dimmed drastically by 96% [7].

Loss to follow-up (LTFU) is a significant cause for treatment failure and threatens the enhancement of HIV treatment outcomes among patients on ART [8]. LTFU is defined as clients stopped ART follow-up for 3 months or longer due to different reasons [2]. At the end of 2014, LTFU was estimated to be 18.7% in Ethiopia, which affects ART effectiveness [9]. This indicates that the expected outcome of ART is achieved when the patients exhibit good adherence with regular follow-up [10].

The incidence of LTFU ranged from 7.1 per 100 person-year in India [11], 10.3 per 100 person-year in South Africa [12], and 43 per 100 person-year in Uganda [13]. In Ethiopia, previous studies reported 8.4 per 100 person-year in Gondar Teaching Hospital [14] and 8.2 per 100 person-year in Aksum St. Marry Hospital [15]. Socio-demographic characteristics [11, 12, 15–19], tuberculosis, ionized prophylaxis, ART side effects, changing ART, duration on ART, viral load, CD4 count, WHO stages III & IV, being bed-ridden, and ambulatory patient were some of the factors associated with LTFU [12–16, 20–22].

Mortality, after loss to follow-up in African countries, ranges from 12 to 87% [23]. LTFU also increased the risk of HIV-related morbidity, drug resistance, hospitalization, and the risk of transmitting drug-resistant strains and shortened the survival status of HIV patients [2, 20, 24]. To tackle these adverse effects, scale-up of ART centers and tracing back of patients as interventions have also been taken from locally and globally [2, 8, 25].

Currently, the Ethiopian government targeted 90% HIV viral load suppression rate for people on ART care and treatment for ending the AIDS epidemic by 2020 [8]. To succeed in this plan, data related to the incidence and predictors of LTFU are crucial. Thus, we conducted this study to estimate the incidence and predictors of LTFU among adult HIV patients on ART at Debre Markos Referral Hospital. The results of this study will inform policymakers and program planners working at various levels of HIV/AIDS control programs.

## Methods

### Study design, setting, and period

A hospital-based retrospective cohort study in Debre-Markos Referral Hospital (DMRH) at Debre Markos Town was undertaken. Debre Markos Town is located 299 km far from Addis Ababa, the capital city of Ethiopia, and 265 km far from Bahir-Dar, the capital city of Amhara Regional State. Debre Markos referral hospital serves more than 3.5 million people of the East Gojjam Administrative Zone and neighboring areas. More than twelve thousands of HIV-positive adults have been initiated antiretroviral therapy (ART) care and treatment in Debre Markos Referral Hospital since 2005. As 2018 annual report, the hospital was providing ART care and treatment services to 3716 adult HIV patients

### Population

All adult HIV patients initiated on ART from January 30, 2008, to January 26, 2018, at Debre Markos Referral Hospital in the ART clinic were our source population. All adult HIV patients initiated on ART from January 30, 2008, to October 30, 2017, at Debre Markos Referral Hospital in the ART clinic were our study population. Therefore, those adult HIV patients who had at least 1-month follow-up after ART initiation were included. On the other hand, we excluded HIV patients who had incomplete baseline records, whose medical charts were not available during the data collection period and patients transferred to Debre Markos referral hospital.

### Sample size determination and sampling procedures

The minimum required sample size was calculated using a survival analysis formula. We used a STATA^TM^ Version 14.2 statistical software to calculate the sample size by considering the following assumptions: the level of significance (*α*) = 5%, *Z*_a/2 (_value at 95% confidence interval = 1.96), power of 80%, and hazard ratio (HR = 2.05 taken from a previous study) [26]. We applied the sample size calculation for each predictor variable, and the one with the maximum sample size (484) was selected for this study. Then, we randomly selected 484 HIV-positive patient records using a computer-generated number among those initiated treatments from January 30, 2008, to January 26, 2018. To know the final status (event or censored) of the patient, we followed the selected records for 10 years or until the end of the study period.

### Variables

The outcome variable was the time to loss to follow-up, whereas the predictors were socio-demographic characteristics (namely, age, sex, marital status, residence, educational, and occupation). Baseline clinical and laboratory characteristics (i.e., duration of ART, adherence, drug side effects, regimen changes, INH prophylaxis, WHO clinical stage, functional status, baseline CD4 counts, and baseline ART regimen).

### Operational definitions

This study aimed to ascertain the incidence of loss to follow-up using 10-year data retrieved from medical record charts. In this study, loss to follow-up was defined as not taking ART refill for 3 months or longer from the last attendance for refill and not yet categorized as dead or transferred-out [27]. ART adherence was defined as the percentage of ART drug dosage calculated from a monthly total dose, and it was classified as good, fair, or poor. Hence, good adherence was reported if equal to or greater than 95% or ≤ 3 dose missing per month, fair if 85–94% or 4–8 dose missing per month, or poor if less than 85% or ≥ 9 dose missing per month [28]. When the patients missed their ART follow-up for three or longer appointment months were considered as an event while those who died during their follow-up, transferred to other health institutions, and remain on treatment at the end of the study period were treated as censored. Furthermore, transfer out was considered as clients who had a transfer letter to initiate ART care and treatment and need to shift after receiving the service. In this study, death was ascertained when HIV patients were documented as death in the ART care and treatment follow-up sheet by the health care professionals.

### Data collection tool and procedure

The data extraction checklist was prepared from the ART entry and follow-up forms. To ensure data quality, before data extraction, the data extraction tool was prepared with care from ART intake and follow-up forms. In addition, we verified consistency between data recording systems and the prepared checklist by randomly selecting and completing 48 sample chart reviews, which resulted in slight amendments to the data extraction checklist. Two BSc nurses who have been working in the ART clinic of Debre Markos Referral Hospital collected the data. Two days of training were given for both data collectors and supervisors to standardize and agree on the way to review medical records. The supervisor and principal investigators supervised the data collectors throughout the entire data collection process.

### Data processing and analysis

Data were entered into Epidata^TM^ Version 4.2 and analyzed using STATA™ Version 14.0 statistical software. At the end of the data extraction period, the outcome of each study subject was dichotomized into censored or event (LTFU). Moreover, to identify the predictor variables, a cox-proportional hazard regression model was fitted. To check the assumption of the Cox-proportional hazard regression model, we used Schoenfeld residual test for continuous variables and Log-Log plot for categorical variables.

Furthermore, we checked the model fitness using a Cox-Snell residual test (see supplementary file fig. 1.). We used the Nelson-Aalen cumulative hazard curve to estimate the occurrence of time to loss to follow-up. A log-rank test was estimated to compare the survival curves for different categorical explanatory variables.

In univariate analysis, we used the mean with standard deviations to describe normally distributed continuous data and median with interquartile range for skewed continuous data. In another way, the categorical data extracted from HIV-positive patients were described using frequency distribution or percentages. Regarding bivariable analysis, the outcome variable (loss to follow-up) and explanatory variables were entered cox-proportional hazard regression model to select variables for the multivariable cox-proportional hazard regression model. As a result, variables having *p* value ≤ 0.25 in the bi-variable analysis were fitted into the multivariable cox-proportion regression model. Finally, the adjusted hazard ratio with its corresponding 95% confidence interval (CI) was used to declare the presence of a significant association between the explanatory and outcome variables.

## Results

### Socio-demographic characteristics

About 484 HIV-positive patient record charts were retrieved, giving a 100% response rate. The median age of the study participants was 32 years with an inter-quartile range (Q1, Q3: 27, 39.5). Two hundred seventy-two (56.20%) of the study participants were from the urban residence. The majority, 406 (83.75%) of HIV-positive patients were married while slightly more than two-thirds (69.21%) of the study participants attended formal education. The majority, 427 (88.41%) of the retrieved HIV-positive patients’ information were Orthodox Christian by religion. During their recruitment for ART, the majority, 420 (86.78%) of the study participants had cell phones. Slightly more than half, 267 (55.17%) of the participants had disclosed their serostatus when they were recruited (Table 1).

**Table 1:** Socio-demographic characteristics of HIV-positive adult individuals on ART at Debre Markos Referral Hospital who were enrolled from January 30, 2008, to September 26, 2017

Characteristics	Frequency	Percentage
Sex		
Male	202	41.74
Female	282	58.26
Age categories		
15–24	53	10.95
25–34	210	43.39
35–44	149	30.79
≥ 45	72	14.88
Education status		
Not formally educated*	149	30.79
Primary education	130	26.86
Secondary education	129	26.65
Tertiary education	76	15.70
Residence		
Urban	272	56.20
Rural	212	43.80
Marital status		
Single	78	16.12
Married	226	47.31
Divorced	114	23.55
Widowed	56	11.57
Separate	7	1.45
Religion		
Orthodox	427	88.22
Muslim	57	11.78
Cell phone		
Yes	420	86.78
No	64	13.22
Disclosure status		
Yes	267	55.17
No	217	44.83

### Baseline clinical characteristics

Two-hundred ninety-three (60.53%) of the study participants were WHO clinical stages of III and IV. Regarding functional status, about three-fourth, 363 (75%) of the study, participants were experiencing working status. The median CD4 count was 161 cells/ml with an inter-quartile range (Q1, Q3: 84, 274.5). Nearly half, 239 (49.38%) of the study participants were taking TDF-3TC-EFV ART regimen. In terms of nutritional status, nearly two-thirds, 319 (65.91%), were well nourished (Table 2).

**Table 2:** Baseline clinical characteristics of HIV-positive adults on ART at Debre Markos Referral Hospital who were enrolled from January 30, 2008, to September 26, 2017

Variables	Frequency	Percentage
WHO stage		
Stage I	117	24.17
Stage II	74	15.29
Stage III	220	45.45
Stage IV	73	15.08
Functional status		
Working	363	75.00
Ambulatory	105	21.69
Bedridden	16	3.31
CD4 count		
≤ 50	57	11.78
50–150	153	31.61
150–200	65	13.43
≤ 200	209	43.18
ART regimen		
d4t-3TC-NVP	64	13.22
d4t-3TC-EFV	61	12.60
AZT-3TC-NVP	80	16.53
AZT-3TC-EFV	40	8.26
TDF-3TC-EFV	239	49.38
Nutritional status		
Well nourished	319	65.91
Not well nourished	165	34.09

### Follow-up characteristics

This study computed that the median follow-up time for the included HIV-positive patients 56 months with an inter-quartile range (Q1, Q3: 12.99, 97.12). The majority 441 (91.12 %) of the participants had a good ART adherence throughout the follow-up period. Almost two-thirds (67.56%) of the study participants had no experience of regimens change. Slightly less than one-third, 151 (31.46%) of the study participants had developed tuberculosis infection during the follow-up period (Table 3).

**Table 3:** Follow-up data of HIV-positive adults on ART at Debre Markos Referral Hospital who were followed from January 30, 2008, to January 26, 2018

Variables	Frequency	Percentage
Duration on ART in months		
3–15	132	27.27
15–27	33	6.82
27–39	32	6.61
39–51	33	6.82
51–63	36	7.44
63–75	34	7.02
75–87	26	5.37
87–99	42	8.68
99–110	61	12.60
110–120	55	11.36
ART adherence		
Good	441	91.12
Fair	33	6.81
Poor	10	2.07
Experience of regimen change		
Yes	157	32.44
No	327	67.56
Experience of TB infection		
Yes	151	31.46
No	329	68.54
Other opportunistic infection		
Yes	258	53.31
No	226	46.69
CPT adherence		
Good	409	84.50
Poor	75	15.50

### Incidence of loss to follow-up

**Fig. 1:**
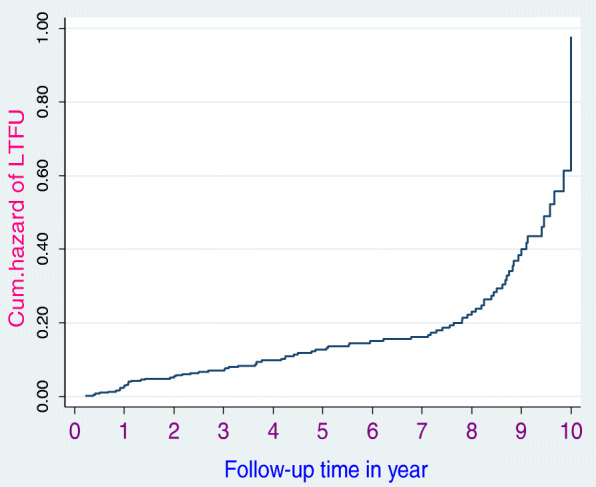
The cumulative hazard rate of lost to ART follow-up among HIV-positive adult individuals at Debre Markos Referral Hospital, North-West Ethiopia, from January 30, 2008, to January 26, 2018

**Fig. 2:**
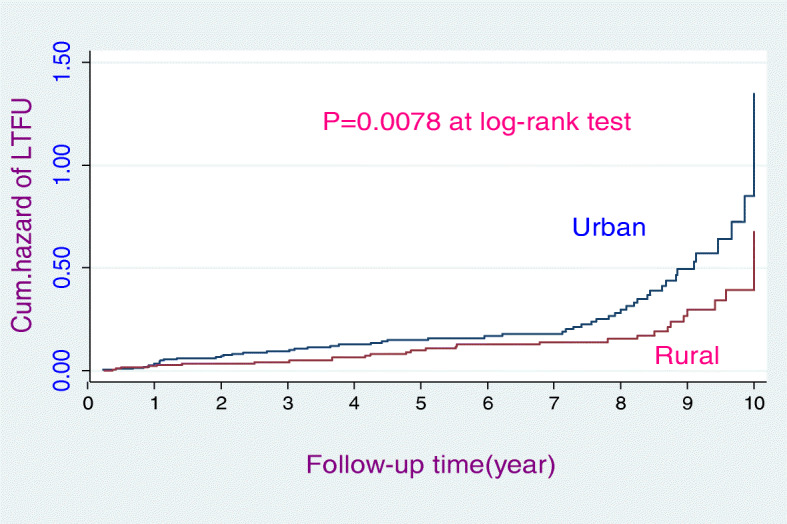
The hazard rate of lost to follow-up of HIV-positive adults on ART over their place of residence from January 30, 2008, to January 26, 2018

**Fig. 3:**
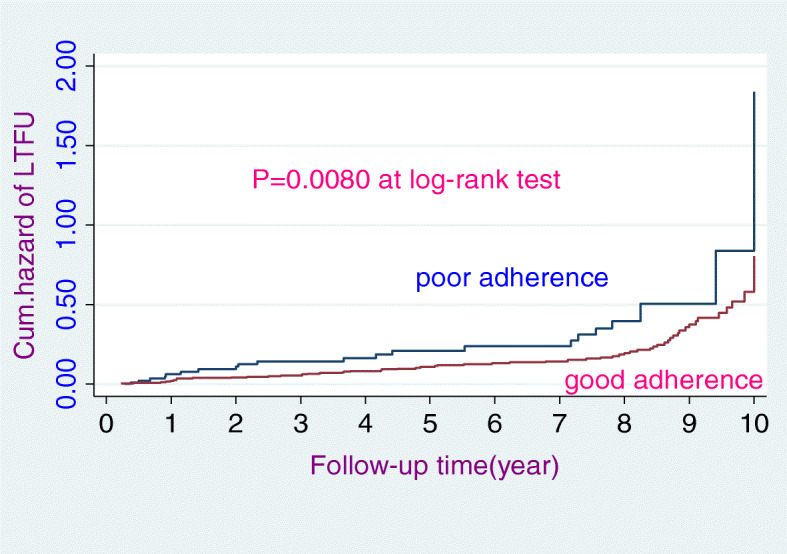
The hazard rate of lost to follow-up of HIV-positive adult individuals on ART over their history of ART adherence from January 30, 2008, to January 26, 2018

**Fig. 4:**
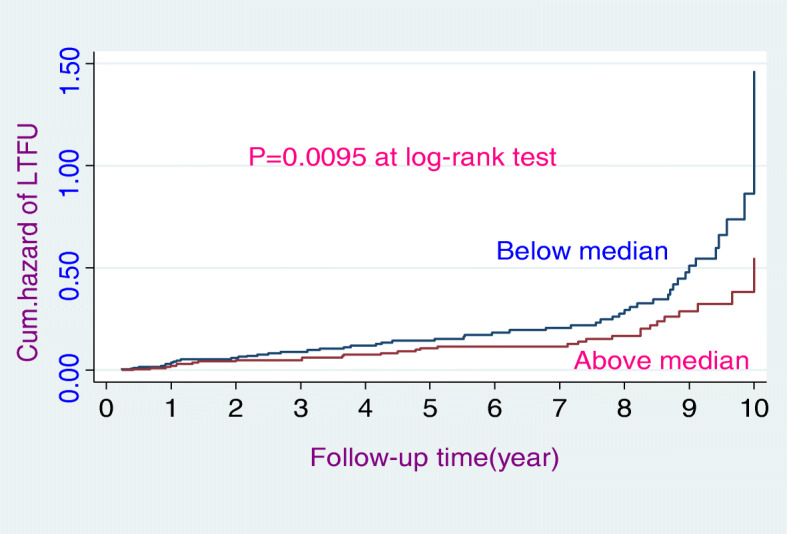
The hazard rate of lost to follow-up of HIV-positive adult individuals on ART over their age categories from January 30, 2008, to January 26, 2018

The study participants had 3–120 months follow-up periods yielding 2294.8 person-years LTFU free time. This study indicated that the proportion of loss to follow-up (LTFU) was 17.36% (*n* = 84). Similarly, the study revealed that the overall incidence rate of loss to follow-up (LTFU) was 3.70 (95% CI: 0.03, 0.05) per 100 person-years. During the 10-year follow-up period, slightly less than one-third (30.95%) of the HIV-positive patients had experienced LTFU. The overall incidence rate (Fig. 1), the incidence rate over residence (Fig. 2), the incidence rate over ART adherence (Fig. 3), and incidence rate over median age (Fig. 4) were compared graphically.

### Bivariable and multivariable cox-regression analysis

During bivariable cox-regression analysis, nine variables, namely, baseline regimen, adherence, WHO clinical stage, residence, having a cell phone, age, baseline CD4 count, experience of TB infection, and experience of regimen change, were found as a candidate for multivariable cox-regression analysis model.

The final multivariable Cox-regression analysis model identified that WHO clinical stage IV, rural residence, and having no cell phones were identified as significant predictors of LTFU. The model fitness was assessed using Cox-snell residual test (see supplementary figure 1). The hazard of loss to follow-up among WHO stage IV HIV patients was 2.8 times (AHR 2.75; 95% CI 1.23, 6.16) higher than WHO clinical stage I HIV patients. The hazard of loss to follow-up among rural residents was decreased by 40% (AHR = 0.60; 95% CI 0.37, 0.99) as compared to urban residents. Furthermore, the hazard of LTFU among individuals who had no cell phone was 1.9 times (AHR 1.90; 95% CI 1.14, 3.40) higher as compared to those who had cell phones (Table 4).

**Table 4:** Multivariable Cox regression analysis of predictors of LTFU among HIV-positive adult subjects on ART at Debre Markos Referral Hospital, North West Ethiopia, from January 30, 2008, to January 26, 2018

Variables	Last status		CHR (95% CI)	AHR (95% CI)	*p* value
	Event	Censored			
Sex					
Male	40	162	1.0	1.0	
Female	44	238	0.81 (0.53,1.23)	0.88 (0.54, 1.42)	0.58
ART regimen					
1a	8	56	1.0	1.0	
1b	13	48	1.6 (0.7, 3.9)	1.6 (0.6, 3.9)	0.37
1c	14	66	1.99 (0.83, 4.8)	2.79 (1.07, 7.23)	0.32
1d	9	31	2.4 (0.94, 6.4)	3.14 (1.13, 8.08)	0.19
1e	40	199	6.7 (3.14, 14.7)	9.3 (3.75 , 23.8)	0.10
History of ART adherence					
Poor	23	77	1.0	1.0	
Good	61	323	0.5(0.3, 0.8)	1.2(0.3, 0.9)	0.20
WHO clinical stage					
Stage I	11	106	1.0	1.0	
Stage II	8	66	0.7 (0.29, 0.1.81)	1.09 (0.42, 2.85)	0.45
Stage III	48	172	1.44 (0.75, 2.78)	1.73 (0.87, 3.46)	0.20
Stage IV	17	56	1.8 (0.83, 3.8)	2.75 (1.23, 6.16)	0.01
Residence					
Urban	55	217	1.0	1.0	
Rural	29	183	0.5 (0.33, 0.8)	0.6 (0.37, 0.99)	0.046
Phone					
Yes	68	352	1.0	1.0	
No	16	48	1.5 (0.87, 2.6)	1.9 (1.14, 3.4)	0.04
Age categories					
15–24	13	40	1.0	1.0	
25–34	41	169	0.44 (.22, 0.88)	0.67 (0.35, 1.29)	0.20
35–44	22	127	0.4 (0.22, 0.86)	0.32 (0.15, 0.67)	0.20
≥ 45	8	64	0.4 (0.17, 0.99)	0.33 (0.13, 0.83)	0.19
Baseline CD4 Count					
≤ 50	11	46	1.0	1.0	
50–150	28	125	0.68 (0.34, 1.37)	0.76 (0.37, 1.57)	0.46
150–200	12	53	0.58 (0.26, 1.33)	0.52 (0.21, 1.28)	0.16
≥ 200	33	176	1.14 (0.57, 2.27)	0.68 (0.32, 1.43)	0.31
History of TB infection					
No	56	273	1.0	1.0	
Yes	25	126	0.58 (0.36, 0.94	0.74 (0.43, 1.26)	0.27
History of ART regimen change					
No	54	273	1.0	1.0	
Yes	30	127	0.72 (0.46, 1.14)	1.59 (0.9, 2.8)	0.11

## Discussion

Antiretroviral therapy is an intervention to control HIV infection, improving the quality of life and prolonging the survival status of HIV patients. So discontinuation of ART care and treatment threatened the effectiveness of such intervention [8]. This retrospective cohort study aimed to determine the incidence and identify predictors of LTFU among adult HIV patients on ART in Debre Markos Referral Hospital.

In this study, the overall incidence rate of loss to follow-up among HIV-positive adults on ART at Debre Markos Referral Hospital was 3.70 per 100 person-years of observation. It was lower compared with the study findings reported in the University of Gondar Teaching Hospital [14], and Aksum St. Hospital, Ethiopia [14], in Asian Pacific, India, and South Africa [11, 12, 21]. The potential elucidation for the disparity might be due to the difference in the study period, commencing of test and treat ART initiation criteria, the difference in the demographic pattern of patients, 1j(TDF-3TC-DTG) ARV used today instead of 1e(TDF-3TC-EFV), change in disease staging criteria, the presence of case manager and adherence supporter and adapting mass laboratory screening criteria [2, 29, 30]. An improvement on ART adherence, tracing back of HIV patients who loss there follow-up to ART, and early initiation of ART have been adapted recently to get expected clinical outcomes and improve the quality of life ultimately [31]. Evidence also suggests that early ART initiation helps to retain HIV-positive patients on care and treatment [32–34].

In this study, the proportion of HIV patients with clinical stages III and IV were lower (60.53%) than studies done previously (77.7% and 84%) [14, 15]. Similarly, in this study, a higher proportion (75%) of the participants were working in functional status as compared with the previous studies (50.96% and 64%) [14, 15]. Hence, the difference in the clinical-stage and functional status might be a reason for loss to follow-up to be lower in this study [14, 15]. Another possible explanation might relate to patients experienced less (32.44%) proportion of drug toxicity in this study as compared with the previous study( 75.5%) [14] that could decrease the rate of loss to follow-up.

Moreover, the lower incidence rate of loss to follow-up reported by this study might be due to differences in participant’s residence. This means the proportion of urban residents was only 56.2% in this study but 96.1% in a previous study [14]. Urban residents are more prone to self-referral to other health care facilities and not permanently live in the area, relatively. This might contribute to the lower rate of loss to follow-up for this study.

The current study also identified that the WHO clinical stage IV and having no cell phone were found to increase the rate of loss to follow-up.

This study identified WHO clinical stage IV as a predictor of loss to follow-up from ART care and treatment. The risk of experiencing loss to follow-up among HIV-positive patients with WHO clinical stage of IV was 2.8 (AHR 2.75; 95% CI 1.23, 6.16) times higher as compared with WHO clinical stage I. This finding is in line with findings reported in Mizan-Tepi University, Oromia Region, and South Africa [12, 19, 20]. This could be explained by those HIV-positive patients with WHO clinical stage IV were highly experienced an immunological deterioration. Due to this, they could not overcome the challenge they face in care and treatment and had discontinued.

Likewise, this study found that the risk of LTFU among rural residents was reduced by 40% (AHR 0.6; 95% CI 0.37, 0.99) compared with urban residents. It is in accordance with the study conducted in India [35]. This might be due to patients coming from rural areas preferred to have ART follow-up in the nearby hospitals. Because rural residents are stable (permanent residents relatively), they did not use the self-referral system to other healthcare institutions due to scarcity of money and they face less stigma.

Furthermore, ART clients who had no cell phones were nearly two (AHR 1.90; 95% CI 1.14, 3.40) times more at risk of experiencing LTFU compared with their counterparts (having a cell phone). This finding might be attributable to the cell phone serving as a means of tracing HIV patients who are on ART. Also, a cell phone may increase the relationship between patients and their health professionals. Cell phones also help to keep an appointment with others and health facilities’ appointment period. This scenario compared well with the previous evidence that cell phone improves health care service delivery processes by targeting communication between providers and their patients. Mobile technologies improve clinical management support in settings where there are no specialist clinicians, and they can be used to send patients test results and timely reminders of appointments [36–38]. Therefore, programmers, communities, associations, stakeholders, planners, government bodies, and health care professionals need to establish strategies to provide a cell phone for those HIV-positive patients with low economic status.

### Limitations of the study

As a limitation, this study was not considered some essential predictors, like viral load, liver, and renal function tests since it was conducted using secondary data that have missing records. In addition to the above, it might lead to underestimating the incidence rate of loss to follow-up owing to record problems.

## Conclusion

This study revealed that the incidence rate of loss to follow-up among adult HIV-positive patients at Debre Markos Referral Hospital was low. Having no cell phone, WHO clinical stage IV, and rural residence were the identified predictors of loss to follow-up. Therefore, program leaders and initiatives should strengthen early initiation (WHO clinical stage I) of ART care and treatment.

## Supplementary information

**Additional file 1: Figure S1.** The goodness of fit test for Cox-proportional hazard regression model using cox-Snell residual.

## Data Availability

Data will be accessed upon request of the correspondence author.

## Abbreviations

AIDS: Acquired immune deficiency syndrome
ART: Antiretroviral therapy
AHR: Adjusted hazard ratio
HIV: Human immunodeficiency virus
PLHIV: People living with human immunodeficiency virus,
WHO: World Health Organization
LTFU: Loss to follow-up
FMOH: Federal Ministry of Health
