# Supplementary material for: Incidence and predictors of loss to follow-up among HIV-positive adults in northwest Ethiopia: a retrospective cohort study
Source: Trop Med Health. 2020 Sep 14;48:78. doi: 10.1186/s41182-020-00266-z (PMC7488994; doi:10.1186/s41182-020-00266-z)
Supplement: Supplementary file 1 — Additional file 1: Figure S1. The goodness of fit test for Cox-proportional hazard regression model using cox-Snell residual. [file 41182_2020_266_MOESM1_ESM.docx]

List of Supplementary Figure legends

Fig.1.The goodness of fit test for Cox-proportional hazard regression model using cox-Snell residual
